# Supplementary material for: Rapid turnover of life-cycle-related genes in the brown algae
Source: Genome Biol. 2019 Feb 14;20:35. doi: 10.1186/s13059-019-1630-6 (PMC6374913; doi:10.1186/s13059-019-1630-6)
Supplement: Supplementary file 3 — Protein alignments, corresponding Gblocks html file and CDS sequences for the lineage conserved gametophyte-biased genes. (ZIP 2109 kb) [file 13059_2019_1630_MOESM3_ESM.zip › Con_GA_alignments/SJ17848.aa_ali.fasta-gb.htm]

SJ17848.aa.fasta.trans.aa\_ali.fasta


## Gblocks 0.91b Results

Processed file: **SJ17848.aa.fasta.trans.aa\_ali.fasta**  
Number of sequences: **2**  
Alignment assumed to be: **Protein**  
New number of positions: **571** (selected positions are underlined in blue)

```
                         10        20        30        40        50        60
                 =========+=========+=========+=========+=========+=========+
SJ17848          PRHTKPKKASGPWLRSSAQAATEGVKVLQHIKIPRNYEAGGGQEEMNGIPRGGQGGGSGG
Mac.32009.1      ----------------------------------RNHWAWGGQKQMNRIPWGGQGGGSAD
                                                   ##########################


                         70        80        90       100       110       120
                 =========+=========+=========+=========+=========+=========+
SJ17848          PAIPPPATSGVGSSATQATKKRGPYRPRLKGKEGAMSSSVDTPRRNAMLSAIARGGETSI
Mac.32009.1      PAIPPPVTSGGGGSATGATKKRRPYKPRLKGKEGAMSSSVDIPKRNAMISTIAGGGESGM
                 ############################################################


                        130       140       150       160       170       180
                 =========+=========+=========+=========+=========+=========+
SJ17848          RRK-GKGKPLGPGTFLPPEAEGRLLLSDPRLIDPAREKKKTSTPTRSYTVEATAFLDIVS
Mac.32009.1      RRKKGKGKPLGTGTIVPHEGQGRLLLSDPKLIHPASVNRKQLTPARSYTVEATAFLDIVS
                 ### ########################################################


                        190       200       210       220       230       240
                 =========+=========+=========+=========+=========+=========+
SJ17848          QVHNRDTDCPANIASDESGAERCDTFNFNCWCKDCGMHFPRLRVAATPTSSVVFGILMAG
Mac.32009.1      QVHNRDTDCPANIASDESGAERCDSFSLNCWCKDCGMQFPRLKVAATPISSVVFGILMAG
                 ############################################################


                        250       260       270       280       290       300
                 =========+=========+=========+=========+=========+=========+
SJ17848          SNIENYCAAHNFSDIADMSRTTYYRELPKVLDAVGQVYDFVMEEQKQAFRKQGWRDVWVD
Mac.32009.1      SNIENYCAAHNFSDIADMSRTTYYRELPKVLDAVGQVFDEVMEGQKEAFREQGWRDVWVD
                 ############################################################


                        310       320       330       340       350       360
                 =========+=========+=========+=========+=========+=========+
SJ17848          TDTLYFSQHKKELEAKGEAFTGIEDGRYENSTPEDMAVGLLLTRKARNDPPAFTEVGGKT
Mac.32009.1      TDTLYFSQHKKELEAKGDAFAGIEEGRYENSTPEDMAVGLLLTRKARNNPPAFTEIAGKT
                 ############################################################


                        370       380       390       400       410       420
                 =========+=========+=========+=========+=========+=========+
SJ17848          LMKGIEVAVDGSYCSRNDMSYSCNVVLSAERSPPNAPDRVTVPFDMVTLTRRTQKDAPLK
Mac.32009.1      LMKGIEVAVDGSYCSRNDMTYLCNVVLSAERSAPDAQDRVTVPFDMVTLSRRTHKDAPLK
                 ############################################################


                        430       440       450       460       470       480
                 =========+=========+=========+=========+=========+=========+
SJ17848          EVCSLLRDGSTCFAHGSEAEWREKVTKGTPREAYKALNVNCYSSSGIRHSLSGQAAEPQG
Mac.32009.1      EVCSLLRDGLTCFAHGSEAEWRKKVSKGTEREAYEALNVNCYSSSGIRHSLRGQAAEPQG
                 ############################################################


                        490       500       510       520       530       540
                 =========+=========+=========+=========+=========+=========+
SJ17848          GRLLCLRLLLSGFYITRFKVANDVGDEMGNLFREFFTEAIELLDWAHVQRDLRCDVAKVA
Mac.32009.1      GRLLCLRLLLSGFYITRFKVANDIGNEMGTLFREFFTEAIELLDWAHVQRDLRCDVAKVA
                 ############################################################


                        550       560       570       580       590       600
                 =========+=========+=========+=========+=========+=========+
SJ17848          PGNGDRIGREFGRAVAEALTESKEEGGLPWENGFVRKFSHITSHFQGDHKYCGQACGGGS
Mac.32009.1      PGNGDRIGRDFGRAVTEALAESKEEGGLPWENGFVRKFAHITSHFQGDHQYCGKDCGGGS
                 ############################################################


                        610       620       630       640       650       660
                 =========+=========+=========+=========+=========+=========+
SJ17848          DDLDPEGRGNASGGVRGKGKGGAAGKISLGDCGTSALTIASARASTEDARSHFEAKQRRM
Mac.32009.1      GYLDSE------------------------------------------------------
                 ######                                                      


                        670       680       690       700       710       720
                 =========+=========+=========+=========+=========+=========+
SJ17848          DEFLTCVEKELQRMTVLGSSEATRSLSALRGSVVFAREKCSEGWSLCNGLVECLEEALCV
Mac.32009.1      ------------------------------------------------------------
                                                                             


                        730       740       750       760       770       780
                 =========+=========+=========+=========+=========+=========+
SJ17848          LEPTVPQESDHARSSLPLSALARVQGNVEGLHSWELASKEGEMGQQRETDGPAYLVTRPD
Mac.32009.1      ------------------------------------------------------------
                                                                             


                        790       800       810       820       830       840
                 =========+=========+=========+=========+=========+=========+
SJ17848          IQEAKLSYVEAVDAVAQAEVLYRAQTKKELAVPVHYMGQTRMGILRQELWCERQSWSFFS
Mac.32009.1      ------------------------------------------------------------
                                                                             


                        850       860       870       880       890       900
                 =========+=========+=========+=========+=========+=========+
SJ17848          HCSSAEGITQRQHHPCQVCDLQAIVQRCSARAFCFCTTSGTHSLDSLNASVSSMTTKKGD
Mac.32009.1      ------------------------------------------------------------
                                                                             


                        910       920       930       940       950       960
                 =========+=========+=========+=========+=========+=========+
SJ17848          FSRSDFAHAQIAVLKRVHPKGQVGMAADVRRKLHLPELSESAAKRVRSREESYSHSKRGE
Mac.32009.1      ------------------------------------------------------------
                                                                             


                        970       980       990      1000      1010      1020
                 =========+=========+=========+=========+=========+=========+
SJ17848          KAARTPSRAVTGEDLKRRRQSGVKKGKAAIQEAMKLNHRAALAGSLAPAGRGKKGEEGDV
Mac.32009.1      ------------------------------------------------------------
                                                                             


                       1030      1040      1050      1060      1070      1080
                 =========+=========+=========+=========+=========+=========+
SJ17848          RNGSELAAGGGRGEGADGGGGGALRGVRTLLANWPLPTGEGESGGGTSGIGAAGEGGGGA
Mac.32009.1      ------------------------------------------------------------
                                                                             


                       1090      1100      1110      1120      1130      1140
                 =========+=========+=========+=========+=========+=========+
SJ17848          GAGAWGGAVGEPPRKSAAFGEAPQRLCQACLGNGVSRSFLAEKAHKRKSQHCEEVQRSWG
Mac.32009.1      ------------------------------------------------------------
                                                                             


                 
                 =======
SJ17848          VQYGYGA
Mac.32009.1      -------
```

```
Parameters used
Minimum Number Of Sequences For A Conserved Position: 2
Minimum Number Of Sequences For A Flanking Position: 2
Maximum Number Of Contiguous Nonconserved Positions: 8
Minimum Length Of A Block: 10
Allowed Gap Positions: None
Use Similarity Matrices: Yes
```

```
Flank positions of the 2 selected block(s)
Flanks: [35  123]  [125  606]  

New number of positions in SJ17848.aa.fasta.trans.aa_ali.fasta-gb:  571  (49% of the original 1147 positions)
```
